# DNA double strand break repair enzymes function at multiple steps in retroviral infection

**DOI:** 10.1186/1742-4690-6-114

**Published:** 2009-12-15

**Authors:** Yasuteru Sakurai, Kenshi Komatsu, Kazunaga Agematsu, Masao Matsuoka

**Affiliations:** 1 Laboratory of Virus Control, Institute for Virus Research, Kyoto University, 53 Shogoin Kawahara-cho, Sakyo-ku, Kyoto 606-8507, Japan; 2 Laboratory of Cell Regulation and Molecular Network, Graduate School of Biostudies, Kyoto University, Kyoto 606-8501, Japan; 3 Department of Genome Repair Dynamics, Radiation Biology Center, Kyoto University, Yoshidakonoe-cho, Sakyo-ku, Kyoto 606-8501, Japan; 4 Department of Infection and Host Defense, Graduate School of Medicine, Shinshu University, 3-1-1, Asahi, Matsumoto, Nagano 390-8621, Japan

## Abstract

**Background:**

DNA double strand break (DSB) repair enzymes are thought to be necessary for retroviral infection, especially for the post-integration repair and circularization of viral cDNA. However, the detailed roles of DSB repair enzymes in retroviral infection remain to be elucidated.

**Results:**

A GFP reporter assay showed that the infectivity of an HIV-based vector decreased in ATM- and DNA-PKcs-deficient cells when compared with their complemented cells, while that of an MLV-based vector was diminished in Mre11- and DNA-PKcs-deficient cells. By using a method based on inverse- and Alu-PCR, we analyzed sequences around 3' HIV-1 integration sites in ATM-, Mre11- and NBS1- deficient cells. Increased abnormal junctions between the HIV-1 provirus and the host DNA were found in these mutant cell lines compared to the complemented cell lines and control MRC5SV cells. The abnormal junctions contained two types of insertions: 1) GT dinucleotides, which are normally removed by integrase during integration, and 2) inserted nucleotides of unknown origin. Artemis-deficient cells also showed such abnormalities. In Mre11-deficient cells, part of a primer binding site sequence was also detected. The 5' host-virus junctions in the mutant cells also contained these types of abnormal nucleotides. Moreover, the host-virus junctions of the MLV provirus showed similar abnormalities. These findings suggest that DSB repair enzymes play roles in the 3'-processing reaction and protection of the ends of viral DNA after reverse transcription. We also identified both 5' and 3' junctional sequences of the same provirus by inverse PCR and found that only the 3' junctions were abnormal with aberrant short repeats, indicating that the integration step was partially impaired in these cells. Furthermore, the conserved base preferences around HIV-1 integration sites were partially altered in ATM-deficient cells.

**Conclusions:**

These results suggest that DSB repair enzymes are involved in multiple steps including integration and pre-integration steps during retroviral replication.

## Background

Integration of viral DNA into the host genome is essential for retroviral replication. In this step, the integrase removes the two terminal nucleotides at each 3' end of the viral DNA (3'-processing) and catalyzes the joining of the processed end to the host DNA (strand transfer) [1]. Since the two ends attack the target DNA in a 5'-staggered fashion, single strand gaps between viral DNA and the target DNA are generated. Host DNA repair enzymes are thought to repair these gaps (post-integration repair). Additionally, unintegrated viral DNA is circularized to form two kinds of circular viral DNAs, 2-LTR circles and 1-LTR circles. Formation of these circular DNAs is also catalyzed by host DNA repair enzymes. Recent studies reported DNA double-strand break (DSB) repair enzymes as candidate catalysts for the post-integration repair and the circularization of viral DNA [2,3].

DSBs are the most serious damage that chromosomal DNA suffers, and must be repaired immediately and appropriately. When DSBs are generated in cellular DNA, ataxia-telangiectasia-mutated (ATM), a major molecular sensor of DSBs, directly binds to the damaged DNA and activates DSB repair pathways by phosphorylating target proteins [4,5]. One of the major targets is the MRN complex, which consists of Mre11, Rad50 and NBS1 [6]. This complex has recently been reported to further enhance ATM activation by recruiting ATM into the damaged site [7-9]. After detecting the damage, ATM activates two DSB repair pathways; homologous recombination (HR), and non-homologous end joining (NHEJ) [10]. In the NHEJ pathway, DNA-dependent protein kinase (DNA-PK), which consists of DNA-PK catalytic subunit (DNA-PKcs) and Ku, binds and holds the two ends of the break together. Then ligase IV/XRCC4/XLF carries out the ligation reaction [11,12]. When the ends are not suitable for direct ligation, Artemis nuclease often processes the ends [13].

Retroviral transduction into mutant cells lacking DNA-PK or ligase IV was reported to induce apoptosis [14-16], suggesting that NHEJ is involved in retroviral replication. Moreover, Lau *et al*. showed that an ATM-specific inhibitor suppressed integration of HIV-1 [17]. These reports support the involvement of DSB repair enzymes in post-integration repair. However, *in vitro *experiments showed only the involvement of the components of the single-strand break repair pathway [18,19]. In addition, some reports showed that DSB repair enzymes were only involved in the circularization of viral DNA [20,21]. However, the observation that Ku binds to retroviral preintegration complex (PIC) raises the possibility that DSB repair enzymes may play other roles in integration or pre-integration steps [20]. Thus, the detailed roles of these enzymes remain to be elucidated.

We report here that defects in DSB repair enzymes enhanced the formation of abnormal junctions between retroviral DNA and the host DNA. Moreover, we observed that the base preferences around HIV-1 integration sites partially changed in ATM-deficient cells. These results indicate that DSB repair enzymes are involved in multiple steps of retroviral replication.

## Results

### Effects of DSB repair enzymes on retroviral transduction efficiency

**Figure 1:**
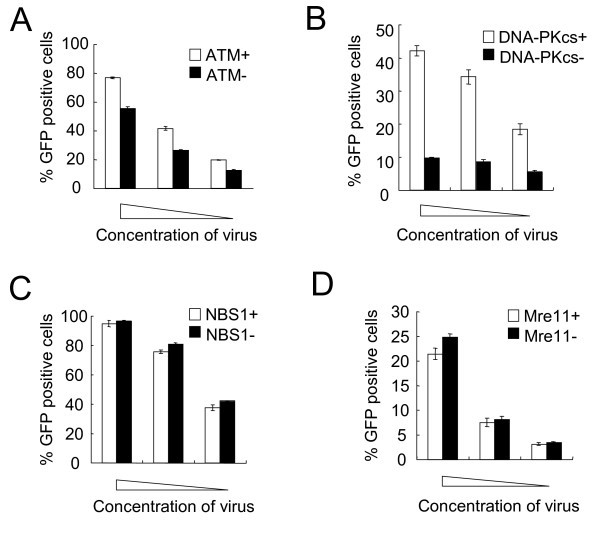
**Transduction efficiency of the HIV-based vector into cells deficient in DSB repair enzymes**. (A) ATM-deficient cells and ATM-complemented cells were transduced with three different dilutions of the HIV-based vector encoding a GFP reporter. Two days postinfection, the percentage of GFP-positive cells was determined by flow cytometry. (B-D) The influence of DNA-PKcs (B), NBS1 (C) and Mre11 (D) on transduction efficiency of the HIV-based vector was investigated by the same method as (A). Error bars represent +/- SD.

Previous reports demonstrated that retroviral infectivity decreased in cells lacking DSB repair enzymes such as ATM and DNA-PKcs [14,16,17]. To confirm whether the enzymes affect HIV-1 infectivity, mutant cell lines and complemented cell lines were transduced with an HIV-based vector encoding a GFP reporter gene. As shown in Figure 1A, the transduction efficiency was impaired in the mutant cells lacking ATM compared to the complemented cells, indicating that ATM is involved in HIV-1 transduction. We also found that DNA-PKcs-deficient M059J cells showed a significantly lower level of transduction efficiency compared to DNA-PKcs-positive M059K cells (Figure 1B), indicating that DNA-PKcs is also required for stable transduction of HIV-1.

The influences of NBS1 and Mre11 on retroviral infectivity were controversial in previous reports [21,22]. In our cell lines, NBS1 and Mre11 deficiencies did not influence transduction efficiency (Figure 1C and 1D), suggesting that the MRN complex might not affect HIV-1 transduction.

We also investigated whether defects in these DSB repair enzymes affected MLV infectivity by using an MLV-based vector encoding a GFP reporter gene. As for the HIV-based vector, the infectivity of the MLV-based vector significantly decreased in DNA-PKcs-deficient cells, indicating the conserved role of DNA-PKcs in retroviral infection (Additional file S1B). Mre11-deficient cells also showed impaired MLV infectivity compared to the complemented cells (Additional file S1D). However, infectivity of MLV vector remained intact in the mutant cells lacking NBS1, which is the other component of the MRN complex (Additional file S1C). This might be due to the different extents of deficiencies of Mre11 and NBS1. In contrast to the HIV-based vector, ATM-deficient cells showed similar transduction efficiency of the MLV-based vector compared to the complemented cells (Additional file S1A). These results suggest that DSB repair enzymes are differentially required for the stable transduction of HIV-1 and MLV.

### Abnormal junctions between HIV-1 provirus and the host DNA in ATM-, Mre11-, NBS1- and Artemis-deficient cells

Since one of the potential targets of DNA repair enzymes is the junction between provirus and the host DNA [18,19,23], we postulated that abnormal junctions would be generated in cells deficient in DNA repair enzymes. We therefore analyzed the sequences of the host-virus junctions. After amplification of integration sites by *Alu *PCR, we used inverse PCR to amplify the sequences around the integration sites with primers specific to LTRs and *Alu *repeat elements [24]. With this method, we could identify integration sites efficiently, with few non-specific amplification products.

**Figure 2:**
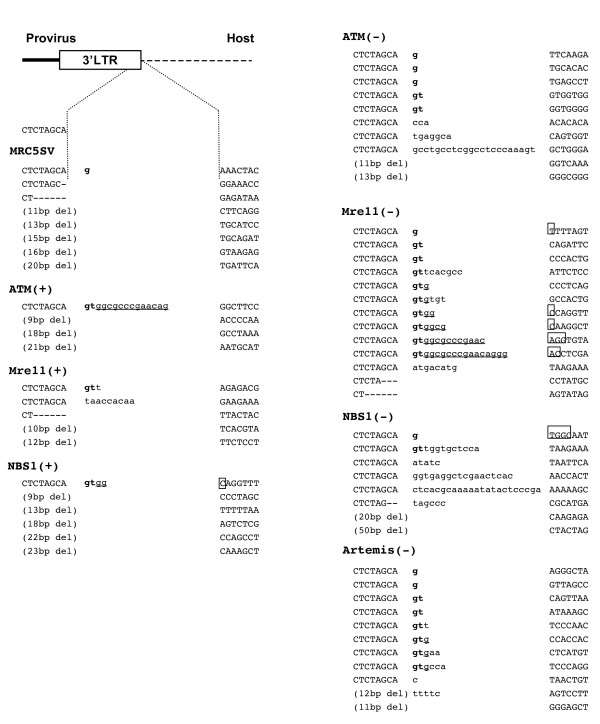
**Abnormal 3' junctions of the HIV-1 provirus in DSB repair enzyme deficient cells**. Junctions between the 3' end of the provirus and the host DNA were analyzed in control cells, mutant cell lines, and complemented cell lines transduced with the HIV-based vector. Inserted abnormal sequences are lowercased. Abnormal nucleotides corresponding to the GT dinucleotides processed by integrase are presented in bold. Partial primer binding site (PBS) sequences are underlined. Squares indicate the location of micro-homologies to the GT dinucleotides and/or PBS.

We analyzed 216 3' junctions between HIV-1 provirus and the host DNA in a control cell line, MRC5SV, and found one abnormal junction with a single nucleotide insertion, and seven junctions with deletions in viral DNA ends (Figure 2). In mutant cells lacking DSB repair enzymes, there were more abnormal junctions with inserted nucleotides between provirus and the host DNA. There were two different groups of abnormal nucleotides. One was a GT dinucleotides (or a G mononucleotide) adjacent to the provirus that is normally removed by integrase in 3'-processing. They did not originate from the host DNA. The other type of abnormal junction contained inserted nucleotides of unknown origin. The number of abnormal junctions with insertions was 1 of 216 (0.5%) events in the control cells, but 8 of 161 (5.0%) events in ATM-deficient cells (Figure 2 and Table 1). In ATM-complemented cells, 1 of 151 (0.7%) junctions had abnormal insertions, which was a significantly lower frequency than that of ATM-deficient cells. Although GFP reporter assays showed that defect of the MRN complex did not affect HIV-1 infectivity, the junctions in the MRN complex deficient cells also had abnormal insertions: 11 of 147 (7.5%) junctions in Mre11-deficient cells and 6 of 145 (4.1%) junctions in NBS1-deficient cells. It is of note that some of the abnormal junctions in Mre11-deficient cells also included 2, 4, 11, or 15 nucleotides of the primer binding site (PBS) sequences (Figure 2). In contrast, abnormal junctions with insertions were less frequent in Mre11-complemented cells (2 of 144: 1.4%) and NBS1- complemented cells (1 of 168: 0.6%). These results indicate that both Mre11 and NBS1 are indeed associated with HIV-1 replication. In contrast, in DNA-PKcs-deficient cells, only 3 of 153 (2.0%) junctions had abnormal insertions (Additional file S2), which is not a statistically significant difference compared to control MRC5SV cells.

**Table 1:** The number of 3' abnormal junctions of the HIV-1 provirus

	ATM(-)	ATM(+)	Mre11(-)	Mre11(+)	NBS1(-)	NBS1(+)	Artemis(-)	MRC5SV
**Insertions**	**8**	**1**	**11**	**2**	**5**	**1**	**9**	**1**
**Insertions + Deletions**	**0**	**0**	**0**	**0**	**1**	**0**	**1**	**0**
Deletions	2	3	2	3	2	5	1	7
Total junctions	161	151	147	144	145	168	136	216

*P *value	0.012		0.023		0.035			
	(0.0046)	(0.80)	(0.00005)	(0.34)	(0.013)	(0.86)	(0.0003)	

The *P *values under the columns of the deficient cell lines are for comparison of the number of junctions with only insertions or both insertions and deletions to that of the corresponding complemented cell lines. The numbers in parentheses under the table represent the *P *values compared to the control MRC5SV cells.

Abnormal junctions with insertions were also found in 10 of 136 (7.4%) junctions in cells deficient in Artemis (Figure 2 and Table 1), which is a target of phosphorylation by ATM and DNA-PKcs [25,26]. Since Artemis-complemented cells could not be established, we could not conclude that these abnormalities observed in Artemis deficient cells were due to the deficiency of Artemis. However, the frequency was much higher than that of control MRC5SV cells (*P *= 0.0003), indicating the potential effects of Artemis on HIV-1 replication.

Some of the abnormal junctions also exhibited micro-homologies in the host sequences, in which 1-4 nucleotides were identical to a part of the GT dinucleotides and/or the PBS sequence following the inserted part (Figure 2). This observation suggests that at least some proviruses with such abnormal junctions might be integrated by a recombination mechanism using these micro-homologies.

### 5' junctional sequences in DSB repair enzymes-deficient cells

**Figure 3:**
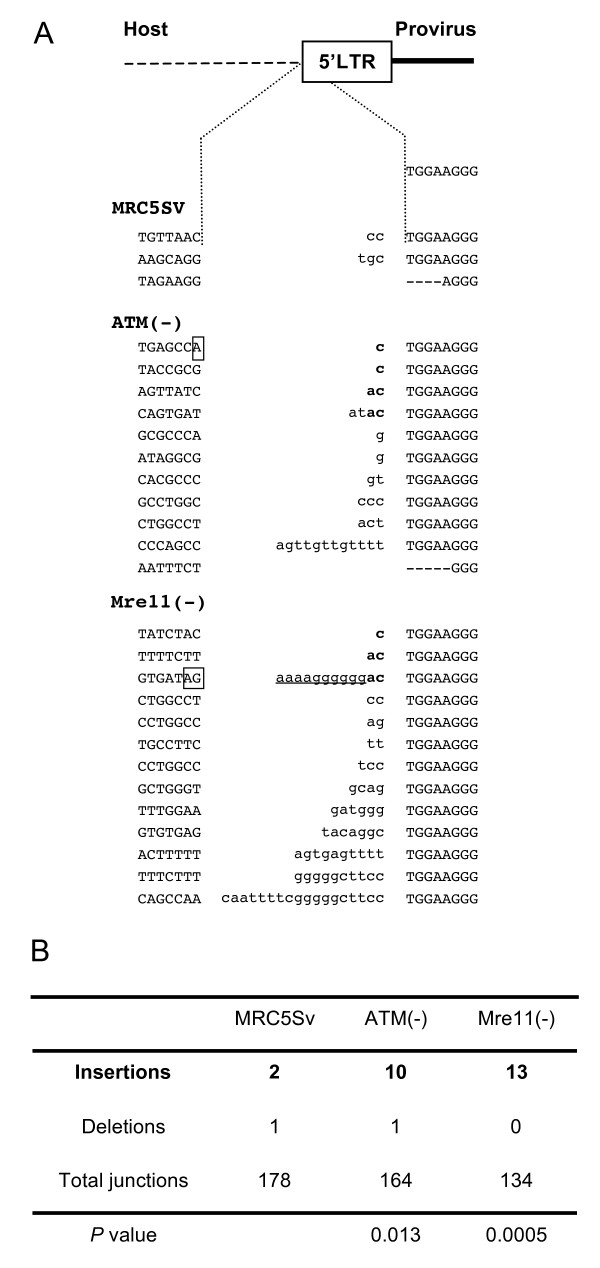
**Abnormal 5' junctions of the HIV-1 provirus in DSB repair enzyme deficient cells**. (A) Junctions between the 5' end of the provirus and the host DNA were analyzed in control and mutant cell lines transduced with the HIV-based vector. Inserted abnormal sequences are in lower case. Abnormal nucleotides corresponding to the sequence (AC) complementary to the GT dinucleotides processed by integrase are presented in bold. Partial polypurine tract (PPT) sequences are underlined. Squares indicate the location of micro-homologies to the AC dinucleotides and/or PPT. (B) The number of junctions with insertions or deletions. The *P *values under the table are for comparison of the number of junctions with insertions in each cell line to that of the control MRC5SV cells.

To investigate whether the abnormalities were common to both ends of provirus, we also analyzed the sequences of 5' junctions. The junctions between the HIV-1 5' LTR and the host DNA also exhibited similar abnormalities (Figure 3A). Abnormal nucleotides were observed in 10 of 164 (6.1%) junctions in ATM-deficient cells and 13 of 134 (9.7%) junctions in Mre11-deficient cells, compared to 2 of 178 (1.1%) junctions in MRC5SV cells (Figure 3B). In 5' junctions, the remaining nucleotides were AC dinucleotides, which are complementary to the GT dinucleotides detected in 3' junctions. In Mre11 deficient cells, 3' polypurine tract (PPT) sequences were also identified. Thus, defects in DSB repair enzymes enhanced the abnormal joining of both ends of the HIV-1 DNA.

### Abnormal junctions of MLV provirus in DSB repair enzyme deficient cells

**Figure 4:**
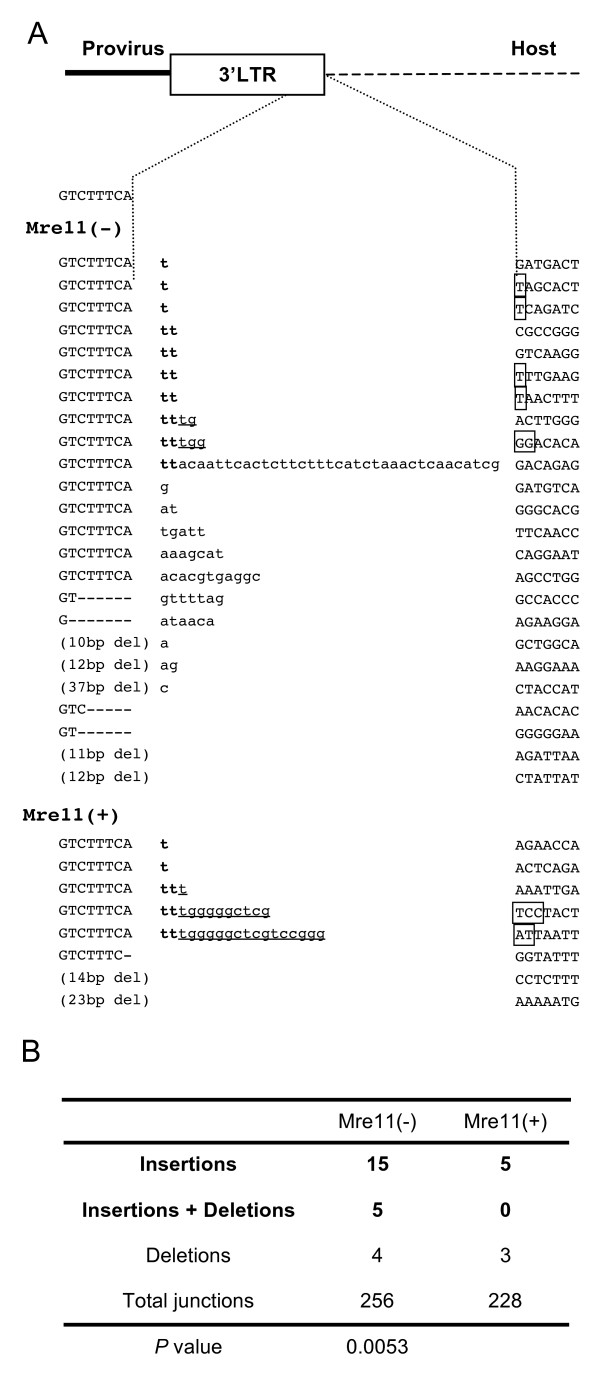
**Abnormal 3' junctions of the MLV provirus in Mre11-deficient cells**. (A) Junctions were analyzed in Mre11-deficient cells and Mre11-complemented cells transduced with the MLV-based vector. Abnormal nucleotides corresponding to dinucleotides (TT) processed by integrase are in bold. Underlined sequences indicate partial PBSs. Squares indicate the location of micro-homologies to TT dinucleotides and/or the PBS. (B) The number of junctions with insertions or deletions. The *P *values under the table are for comparison of the number of junctions with insertions in Mre11-deficient cells to that of Mre11-complemented cells.

To determine whether these abnormalities are specific to HIV-1, we also analyzed sequences of the 3' junctions of the MLV provirus. Junctions with abnormal nucleotides increased from 5 of 228 (2.2%) events in Mre11-complemented cells to 20 of 256 (7.8%) events in Mre11-deficient cells (Figure 4). The abnormal junctions also included TT dinucleotides, which are usually removed by MLV integrase in 3'-processing. Taken together, these results show that defects in DSB repair enzymes increase abnormal host-virus junctions in both HIV-1 and MLV.

### Junctional sequences at the both ends of provirus

**Figure 5:**
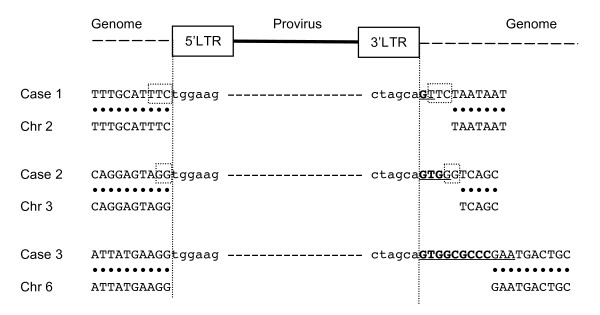
**The 5' and 3' junctional sequences of the same HIV provirus in Mre11-deficient cells**. Junctions between both ends of HIV provirus and the host DNA were analyzed together in Mre11-deficient cells transduced with the HIV-based vector. Three cases including abnormal junctions are shown. In each case, the integrated HIV provirus (top) and the host genome (bottom) are compared. Proviral sequences are in lower case. Inserted abnormal nucleotides are shown in bold. The GT dinucleotides and primer binding site (PBS) sequences are underlined. Squares indicate short repeats flanking the provirus.

To study whether both 5'- and 3'-junctions of the same provirus were abnormal, we analyzed both 5' and 3' junctional sequences of the same provirus. Since the method used in Figure 2, 3 and 4 could detect only one end of provirus, we next adopted a traditional inverse PCR method. We identified three HIV-1 proviruses with abnormal junctions in Mre11-deficient cells (Figure 5). All three proviruses had the abnormal nucleotides at the 3' junctions. A single G was inserted in case 1, while both GT dinucletotides and part of a PBS were inserted in cases 2 and 3. These 3' junctions also showed micro-homologies in the host sequences, confirming the abnormalities shown in Figure 2. However, the 5' junctions were intact in these proviruses, indicating that these 5' junctions were processed by integrase as per normal. We also found that the host sequence adjacent to the provirus contained short repeats in case 1 and 2. Although all of the other proviruses had 5-bp short repeats as reported previously (data not shown), case 1 and 2 contained 3-bp and 2-bp short repeats, respectively. Case 3 lacked short repeats. These results suggest that the integration of these proviruses was catalyzed by integrase, but in abnormal ways.

### Altered base preference surrounding HIV-1 integration sites in cells lacking ATM

**Figure 6:**
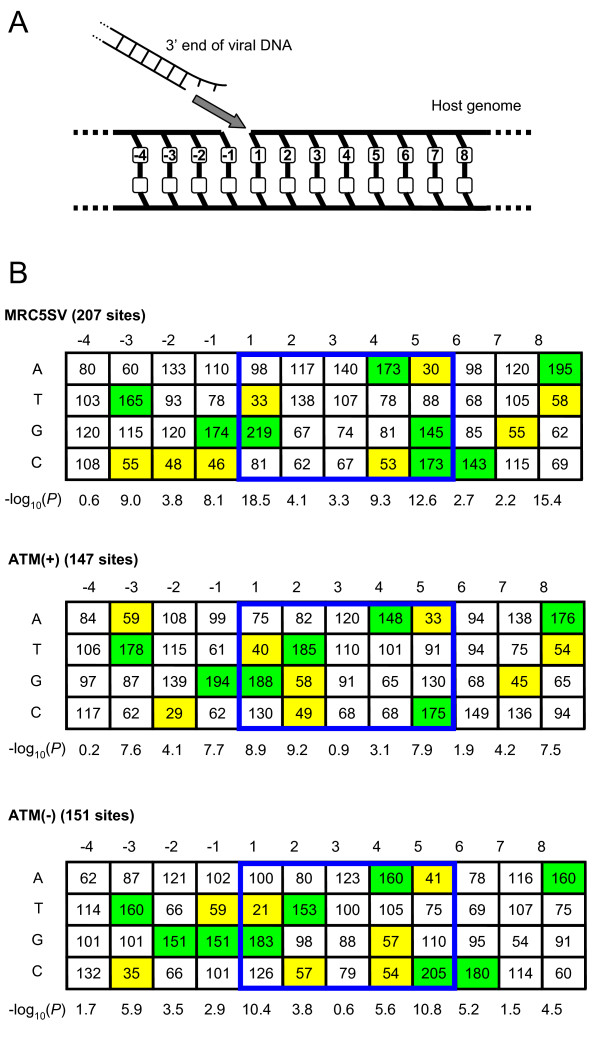
**The local base preferences surrounding 3' ends of HIV-1 proviruses integrated in ATM-deficient cells**. (A) A schematic figure of the strand transfer reaction of HIV-1. The 3' end of viral DNA attacks the phosphodiester bond between positions -1 and 1 of the host DNA, and covalently joins to the position 1 nucleotide. (B) Base compositions around the integration sites in the control MRC5SV cells, ATM-complemented cells and ATM-deficient cells. The sequences represent the target DNA sequence before the viral DNA is inserted between the position 1 and -1. The 5 bp sequences (positions 1 to 5), which are duplicated next to both ends of the provirus, are boxed by blue lines. Each tabulated number represents the observed base frequency divided by the expected base frequency at each position. The expected base frequencies are average frequencies observed in human genome (A:29%, T:29%, G:21%, C:21%). The P values are obtained by χ^2 ^analysis comparing observed and expected base compositions at each position. At the positions with *P *< 0.01, frequencies < 60% and frequencies > 140% of expected frequencies are colored yellow and green, respectively.

**Figure 7:**
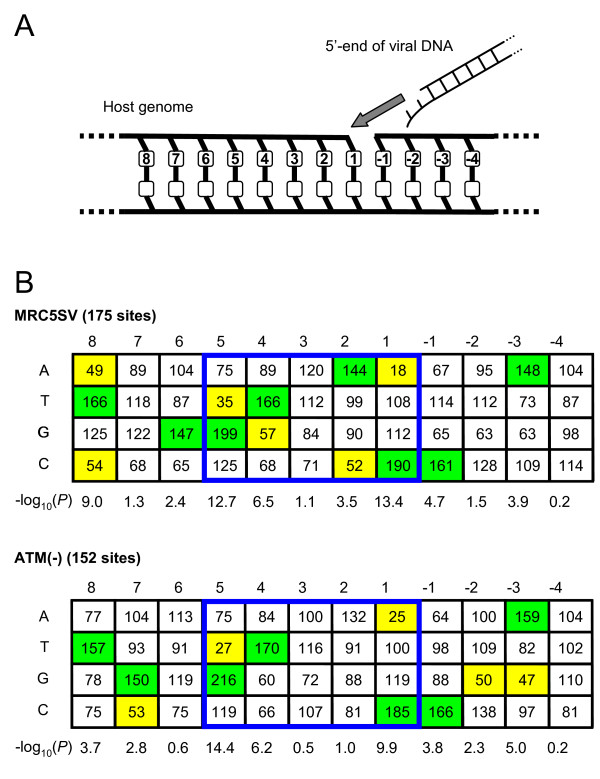
**The local base preferences surrounding 5' ends of HIV-1 proviruses integrated in ATM-deficient cells**. (A) A schematic figure of the strand transfer reaction of HIV-1. The 5' end of viral DNA attacks the phosphodiester bond between positions -1 and 1 of the host DNA, and covalently joins to the position 1 nucleotide. (B) Base compositions around the integration sites in the control MRC5SV cells and ATM-deficient cells. The sequences represent the target DNA sequence before the viral DNA is inserted between the position 1 and -1. The 5 bp sequences (positions 1 to 5), which are duplicated next to both ends of the provirus, are boxed by blue lines. Each tabulated number represents the observed base frequency divided by the expected base frequency at each position. The expected base frequencies are average frequencies observed in the human genome (A:29%, G:21%, C:21%). The P values are obtained by χ^2 ^analysis comparing observed and expected base compositions at each position. At the positions with *P *< 0.01, frequencies < 60% and frequencies > 140% of expected frequencies are colored yellow and green, respectively.

Retrovirus-specific base preferences in the immediate vicinity of integration sites have been reported [27-29]. Our findings of abnormal host-virus junctions prompted us to investigate whether deficiencies in DSB repair enzymes also influence these preference patterns. We analyzed the nucleotide frequencies for the 8 nucleotides downstream and the 4 nucleotides upstream of the 3' ends of HIV-1 proviruses without insertions and/or deletions (Figure 6B). As shown in Figures 6 and 7, we calculated P values at each position by χ^2 ^analysis comparing the base compositions in each cell line and the average base compositions in the human genome (A:29%, T:29%, G:21%, C:21%). At the positions with *P *< 0.01, the bases with high frequencies or low frequencies were focused and colored in Figure 6 and 7. Compared to the control MRC5SV cells and ATM-complemented cells, which showed a preference pattern similar to that in the previous report [28], ATM-deficient cells showed a partially altered pattern. In the position -2, the different patterns were found in ATM-deficient cells compared to control MRC5SV cells (*P *< 0.0001) or ATM-complemented cells (*P *< 10^-14^). Especially, ATM-deficient cells showed higher frequency of G compared to the control MRC5SV cells and the complemented cells at the position -2. Similarly, integration sites for the 5' end of the provirus in ATM-deficient cells showed a changed preference pattern in position 7 compared to the control MRC5SV cells (*P *< 0.001), in which ATM-deficient cells showed a higher frequency of G (Figure 7B). Since the 5 bp sequence (positions 1 to 5) is duplicated next to the 3' and 5' ends of the provirus as short repeats, position 7 for the 5' end of the provirus corresponds to position -2 for the 3' end of the provirus. This indicates that the analyses at both ends of the provirus showed the same change, suggesting the influence of deficiency in ATM in the position. In contrast, NBS1- and Mre11-deficient cells showed no clear change in base preference (data not shown). Thus, deficiency in ATM partially influences the local base preference pattern surrounding HIV-1 integration sites.

### Effects of the MRN complex on circularization of HIV-1 cDNA

**Figure 8:**
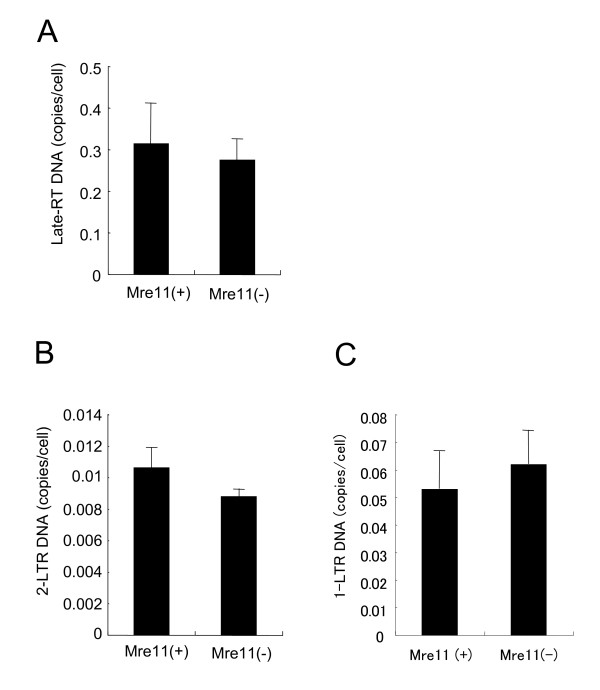
**Quantification of viral cDNA in Mre11-deficient cells and the complemented cells**. Mre11-deficient and complemented cells were transduced with the HIV-based vector, and the total DNA was extracted. By fluorescent-monitored quantitative PCR, total viral DNA (A), 2-LTR circles (B) and 1-LTR circles (C) were quantified. Error bars represent +/- SD.

Previous reports suggested that some DSB repair enzymes were involved in the formation of 2-LTR circles and 1-LTR circles [20,21]. To investigate whether the formation of abnormal host-virus junctions links to circularization of viral cDNA, we quantified total viral cDNA, 2-LTR circles and 1-LTR circles in Mre11-deficient cells and the complemented cells. Quantitative analyses of these viral cDNAs showed that the amount of all three types of viral cDNA was similar in the deficient cells and the complemented cells (Figure 8). This suggested that deficiency in the MRN complex did not influence the formation of viral circular DNAs at least in these cell lines.

## Discussion

This study revealed that deficiencies in some DSB repair enzymes caused abnormalities surrounding retroviral integration sites. Although the GFP reporter assay indicated involvement of ATM and DNA-PKcs in HIV-1 infection consistent with previous reports [14,16,17], the sequence analyses of the host-virus junctions revealed that Mre11 and NBS1 were also involved in HIV-1 infection. In addition, both the GFP reporter assay and the sequence analysis showed the involvement of Mre11 in MLV infection. These results suggest that DSB repair enzymes are more important in retroviral infection than previously thought.

We found two kinds of abnormal junctions in ATM-, Mre11-, NBS1- and Artemis-deficient cells. One contained remnant dinucleotides, which are normally removed from the ends of viral DNA. These were identical to nucleotides processed in 3'-processing [30], which suggest that integrase could not completely process the terminal dinucleotides, or that the processed 3'-ends were repaired during integration. This abnormality suggests that ATM, the MRN complex and Artemis play roles in the 3'-processing activity of integrase and possibly the protection of the ends of viral DNA before strand transfer. In addition, abnormal junctions containing sequences derived from the PBS were found only in Mre11-deficient cells. As the tRNA primer is thought to be removed by the RNase H domain of reverse transcriptase (RT) [31,32], Mre11 may regulate RT to cleave the tRNA correctly. It is noteworthy that a part of 3' PPT sequence of HIV-1, which is a primer sequence for the synthesis of the plus strand, was found at 5' junctions in Mre11 deficient cells. Inserted aberrant nucleotides of unknown origin were another junctional abnormality. Considering that one strand of viral DNA has already bound to the host DNA in the integration intermediate, it is likely that the inserted nucleotides were added at the viral DNA ends before strand transfer. It has been demonstrated that ATM and the MRN complex protect human telomeres, by capping them [33,34]. In addition, a report regarding telomere instability in Artemis-deficient cells suggests that Artemis also protects telomeres [35]. Given that telomeres and unintegrated retroviral DNA ends are similar, DSB repair enzymes including ATM, the MRN complex and Artemis may protect the ends of unintegrated viral DNA from aberrant nucleotide addition.

One reason for the inconsistency between the GFP reporter assay and the sequence analyses, particularly in Mre11 and NBS1, may be that the frequencies of the abnormalities at the host-virus junctions were low. Therefore, it was not detected by the GFP reporter assay. In addition, the GFP reporter assay could detect integrated provirus with abnormal junctions. Therefore, the GFP assay could not discriminate provirus with abnormal junctions from normally integrated provirus. It is possible that the integration efficiency of viral DNA with abnormal ends might be low compared with normal viral DNA, which might underestimate the frequencies of provirus with aberrant ends. Since the deficiencies of Mre11 and NBS1 in the mutant cell lines were reported to be only hypomorphic, the effects of their deficiencies are likely limited in this study [36]. However, the finding that the insertional abnormalities were more frequent in the deficient cell lines compared to the control cell lines indicates the existence of an association between retroviral infection and DSB repair enzymes including Mre11 and NBS1. This was also supported by one of the recent reports that identified host factors by genome-wide screening using an RNAi library [37]. In this report, the knockdown of Mre11 decreased retroviral infectivity.

The identification of the abnormal junctions prompted us to investigate how proviruses with such junctions were integrated. The micro-homologies in the host sequences suggest that integrase-independent recombination is involved in this step (Figure 2, 3 and 4). However, when both 5' and 3' junctional sequences of the same provirus were analyzed, only the 3' junctions of the provirus were abnormal while the 5' junctions were intact (Figure 5), suggesting the involvement of integrase in the establishment of these proviruses. In addition, although normal HIV-1 integration generates 5-bp short repeats flanking the provirus, the abnormal proviruses lacked short repeat or had aberrant (2- or 3-bp) short repeats. These findings suggest that these proviruses were established by impaired activity of integrase.

There are inconsistencies in previous reports regarding the roles of DNA repair enzymes in retroviral replication [38-42]. This is partly because almost all of these studies were based on measuring the retroviral infectivity or apoptosis by retroviral transduction as was done in Figure 1 and S1. Such assays largely depend on the extent of deficiencies or the expression levels of the complemented proteins. The situation is further complicated by the fact that complete deletion of some DSB repair enzymes such as Mre11 and NBS1 is lethal, and there are only hypomorphic mutant cell lines [36]. In some reports, suppressed expression of LEDGF/p75, which is a critical host factor of HIV-1 replication, had no or only modest effect on HIV-1 infectivity [43,44]. However, biochemical assays and sequence analyses in the same cell lines in other studies revealed a strong association of LEDGF/p75 with HIV-1 replication, suggesting that the quantitative assays could not detect all abnormalities [45-47]. Indeed, our sequence analyses revealed abnormalities undetected by the GFP reporter assay in Mre11- and NBS1- deficient cells. These results show the importance of qualitative assays to evaluate the involvement of host factors including DSB repair enzymes in retroviral replication.

Our sequence analyses also showed that deficiencies of DSB repair enzymes influenced HIV-1 integration site selection (Figure 6 and 7). In a recent and substantial effort to understand the mechanism of retroviral integration site selection, Holman *et al*. demonstrated virus-specific base preferences around retroviral integration sites by analyzing massive numbers of integration sites [28]. Our data showing partially altered patterns in ATM-deficient cells reveal that the preference pattern of HIV-1 is marginally influenced by ATM. Interestingly, a lack of ATM caused the appearance of a new base preference. As the new preference may limit the selection of a target DNA sequence, the appearance of the new preference is consistent with decreased HIV-1 infectivity in ATM-deficient cells.

Besides post-integration repair and circularization of viral cDNA, we propose additional possible roles for DSB repair enzymes. Given that Ku was reported to bind to retroviral PICs [20,22], DSB repair enzymes investigated in this study may also bind to PICs and directly regulate their activities. Although further studies are necessary to validate our models regarding the roles of DSB repair enzymes, this study suggests that DSB repair enzymes are involved in retroviral replication in more ways than previously thought. This study sheds light on novel links between DSB repair enzymes and retrovirus, and raises new questions about the detailed mechanism by which DSB repair enzymes control retroviral replication.

## Conclusions

This study showed aberrant sequences surrounding retroviral integration sites in DSB repair enzyme deficient cells; increased abnormal nucleotides at the host-virus junctions and partially altered base preferences surrounding integration sites. These results suggest that DSB repair enzymes are involved in both retroviral integration and pre-integration steps.

## Methods

### Cell lines

293T cells and MRC5SV cells, an SV40-transformed human fibroblast line, were cultured in Dulbecco's modified Eagle's medium (DMEM) and were supplemented with 10% fetal bovine serum, 2 mM L-glutamine, 100 U/ml penicillin, and 50 μg/ml streptomycin. Adenovirus-transformed Artemis-deficient cells originated from RS-SCID patients and were cultured in DMEM [48]. ATM-deficient and ATM-complemented cells were established by transfecting empty vector and ATM expression vector, respectively, into an A-T cell line, AT5BIVA, as described previously [49], and cultured in DMEM containing 200 μg/ml hygromycin B (Calbiochem, San Diego, CA). NBS1-deficient and NBS1-complemented cells were established by transfecting empty vector and NBS1 expression vector, respectively, into an NBS cell line, GM7166VA7, as described previously [50], and cultured in DMEM containing 500 μg/ml G418 (Nacalai tesque, Kyoto, Japan). Mre11-deficient cells were established by transforming an ATLD2 cell line, D6809 (a generous gift from Dr. P. Concannon), by SV40, and the cells were cultured in DMEM. To obtain Mre11-complemented cells, Mre11-deficient cells were transfected with the Mre11 expression vector pCMV-Tag-Mre11, which was created by cloning Mre11 cDNA between the EcoRI and ApaI sites of pCMV-Tag 2B (Clontech, Mountain View, CA), and the cells were cultured in DMEM containing 500 μg/ml G418. For all experiments, we used antibiotic-free medium before 24 h of experiments.

### Production of viral vectors

An HIV-based vector encoding a green fluorescent protein (GFP) reporter was produced as follows. 293T cells were transfected by TransFectin (Bio-Rad, Hercules, CA) with the pCSII-EF-MCS-IRES-hrGFP transfer vector [51], the pCMV-Δ8/9 packaging vector, and pcDNA-VSVG envelope coding vector (generous gift from Dr H Miyoshi, RIKEN, Tsukuba, Japan). Two days after transfection, the supernatant was harvested, passed through a 0.45-μm-pore-size filter, and then subjected to centrifugation at 4°C and 75,000 × *g *for 2 h to concentrate the virus. The virus-containing pellet was dissolved in DMEM.

To produce an MLV-based vector encoding a GFP reporter, the transfer vector pDON-AI-2-IRES-hrGFP was created by excising IRES-hrGFP from pCSII-EF- MCS-IRES-hrGFP via BamHI/HpaI digestion and inserting the DNA into the corresponding site of pDON-AI-2 (Takara Bio, Ohtsu, Japan). GP293 cells, containing a plasmid expressing MLV *gag *and *pol *genes, were transfected with pDON-AI-2-IRES-hrGFP and pcDNA-VSVG. 2 days after transfection, supernatant was harvested, and virus was concentrated.

The titer of these vectors was determined using 293T cells, and scoring of transduction was performed by flow cytometry.

An HIV-based vector encoding a neomycin resistance gene was produced by transfecting the pCMV-Δ8/9 packaging vector, pcDNA-VSVG envelope coding vector, and CSII-CMV-IRES Neo^r^, which was constructed by inserting IRES and a neomycin resistance gene into CSII-CMV-MCS (a generous gift from Dr H Miyoshi, RIKEN, Tsukuba, Japan).

### Single round transduction assay

The mutant cell lines and the complemented cell lines were transduced with various dilutions of the HIV GFP vector or the MLV GFP vector in the presence of 8 μg/ml of polybrene (Sigma, St Louis, MO) for 12 h before changing the medium. The infected cells were harvested two days post-infection and analyzed by flow cytometry to determine the percentage of GFP-expressing cells in each sample.

### Cloning of retroviral integration sites

For cloning of retroviral integration sites by the Alu-PCR-based method, cells transduced with the HIV-based vector for 2 days were collected and the genomic DNA was obtained by standard phenol-chloroform methods with proteinase K treatment. 3' junctional sequences of HIV were amplified by 1st long PCR using a primer (HIV3-1) specific to the U5 region in the HIV LTR and a primer (Alu-1) specific to the Alu repeat sequence. The amplification products were blunted using T4 DNA Polymerase (TOYOBO, Osaka, Japan), phosphorylated using T4 Polynucleotide Kinase (TOYOBO), and circularized and/or concatemerized using T4 DNA Ligase (TOYOBO). The ligation products were amplified by 2nd long PCR using a primer (HIV3-2) specific to the U5 region in the HIV LTR and a primer (HIV3-3) spanning the junctions generated by ligation. Similarly, 5' junctional sequences of HIV were amplified by 1st PCR using a primer (HIV5-1) specific to the U3 region in the HIV LTR and a primer (Alu-2) specific to the Alu repeat sequence, and 2nd PCR using a primer (HIV5-2) specific to the U3 region in the HIV LTR and a primer (HIV5-3) spanning the junctions generated by ligation. 3' junctional sequences of MLV were amplified by 1st PCR using a primer (MLV3-1) specific to the U5 region in the MLV LTR and a primer (Alu-1) specific to Alu repeat sequence, and 2nd PCR using a primer (MLV3-2) specific to the U5 region in the MLV LTR and a primer (MLV3-3) spanning the junctions generated by ligation. The 2nd PCR products were cloned into the pGEM-T Easy Vector (Invitrogen, Carlsbad, CA), which allows for isolation of individual clones.

For cloning of integration sites including 5' and 3' ends of the same provirus, Mre11-deficient cells were transduced by the HIV-based vector encoding a neomycin resistance gene and cultured in DMEM containing 500 μg/ml G418 for a month. After DNA extraction, the genomic DNA was digested with EcoRI, circularized using T4 DNA Ligase, and digested with NotI. Then, both of the junctional sequences of HIV provirus were amplified by 1st long PCR using a primer (HIV-U5) specific to the U5 region in the HIV LTR and a primer (HIV5-1) that was previously described and 2nd long PCR using another primer (HIV3-1) that was previously described and a primer (HIV-U3) specific to the U3 region in the HIV LTR. The 2nd PCR products were cloned into the pGEM-T Easy Vector.

The sequences of the primers used in these assays are described in Additional file S1.

### Sequence analysis of retroviral integration sites

Sequencing was performed using the Big Dye Terminator (version 3.1) cycle sequencing kit and an ABI3130 autosequencer (both from Applied Biosystems, Foster City, CA). The BLAT program http://genome.ucsc.edu, hosted at the University of California, Santa Cruz, was used to search each integration clone against the March 2006 freeze of the human genome. Low-quality sequences and sequences with < 20 base pairs (bp) were discarded.

### Quantification of HIV-1 cDNA

HIV-1 cDNA was quantified by fluorescent-monitored quantitative PCR (Taqman) with an ABI Prism 7700 sequence detection system (Applied Biosystems) essentially as described [24]. Cells were infected with the HIV-based vector and the total DNA was extracted with DNAzol (Invitrogen) after 12 h or 24 h for analysis of total cDNA or 2-LTR and 1-LTR circles, respectively. Sequences of primers and probes are as follows; total cDNA forward, late RT F: 5'-TGTGTGCCCGTCTGTTGTGT-3'; total cDNA reverse, late RT R: 5'-GAGTCCTGCGTCGAGAGAGC-3'; total cDNA probe, LRT-P: 5'-(FAM)-CAGTGGCGCCCGAACAGGGA-(TAMRA)-3'; 2-LTR circle forward, 2-LTR F: 5'-AACTAGGGAACCCACTGCTTAAG-3'; 2-LTR reverse, 2-LTR-R: 5'-TCCACAGATCAAGGATATCTTGTC-3'; 2-LTR probe, MH603: 5'-(FAM)-ACACTACTTGAAGCACTCAAGGCAAGCTTT-(TAMRA)-3'; 1-LTR circle forward, 1-LTR F: 5'-CACACCTCAGGTACCTTTAAGA-3'; 1-LTR reverse, 1-LTR-R: 5'-GCGCTTCAGCAAGCCGAGTCCT-3'; 1-LTR probe, MH603: 5'-(FAM)-ACACTACTTGAAGCACTCAAGGCAAGCTTT-(TAMRA)-3'. Under our PCR conditions with 1-LTR-F and 1-LTR-R primers, 1-LTR circle products (~660 bp) were preferentially amplified compared with 2-LTR circle products (~1170 bp), as described previously [52]. This was verified by checking the specific amplicon generated by standard PCR with the same conditions. For standard curves, we constructed control plasmids by PCR amplification from the total DNA extracts using the same primers as fluorescent-monitored quantitative PCR and cloning the products into the pGEM-T Easy Vector.

## Competing interests

The authors declare that they have no competing interests.

## Authors' contributions

YS and MM designed and performed research; KK and KA contributed new reagents/analytic tools; YS, KK, and MM analyzed data; YS and MM wrote the paper.

## Supplementary Material

Additional file 1 **Figure S1**. Transduction efficiency of an MLV-based vector into cells deficient in DSB repair enzymes. Description: The transduction efficiency of the MLV-based vector was drastically decreased in DNA-PKcs-deficient cells and decreased in Mre11-deficient cells, but not altered in ATM- and NBS1-deficient cells. (A) ATM-deficient cells and ATM-complemented cells were transduced with the MLV-based vector encoding a GFP reporter. 2 days postinfection, the percentage of GFP-positive cells was determined by flow cytometry. (B-D) The influence of DNA-PKcs (B), NBS1 (C) and Mre11 (D) on transduction efficiency of the MLV-based vector was investigated by the same method as in (A). Error bars represent +/- SD. Click here for file

Additional file 2 **Figure S2**. Abnormal 3' junctions of the HIV-1 provirus in DNA-PKcs-deficient cells. Description: (A) Junctions between the 3' end of the provirus and the host DNA were analyzed in DNA-PKcs-deficient cells transduced with an HIV-based vector. Inserted abnormal sequences are in lower case. Abnormal nucleotides corresponding to the GT dinucleotides processed by integrase are presented in bold. (B) The number of junctions with insertions and/or deletions. The *P *values under the table are for comparison of the number of junctions with only insertions or both insertions and deletions to that of MRC5SV cells in Table 1. Click here for file

Additional file 3 **Table S1**. Primers for the sequence analyses around retroviral integration sites. Click here for file
