# Supplementary figures and images for: DNA double strand break repair enzymes function at multiple steps in retroviral infection
Source: Retrovirology. 2009 Dec 15;6:114. doi: 10.1186/1742-4690-6-114 (PMC2797772; doi:10.1186/1742-4690-6-114)

**A**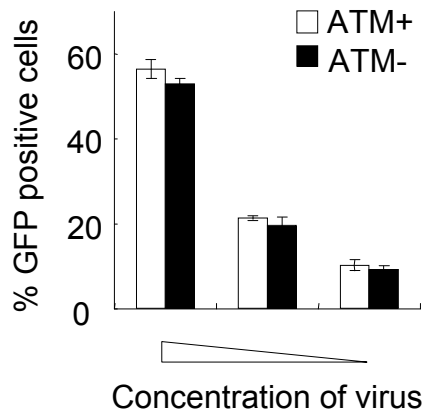**B**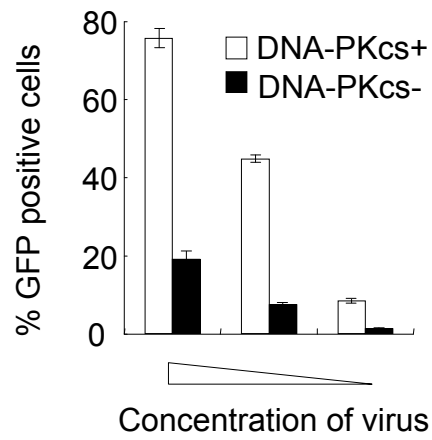**C**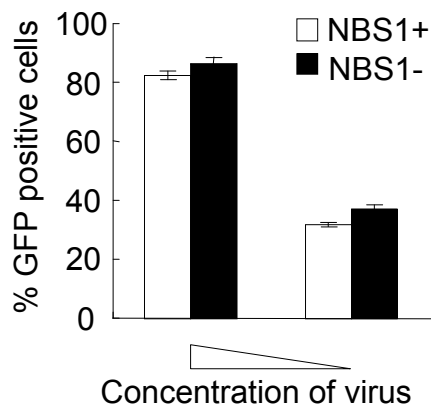**D**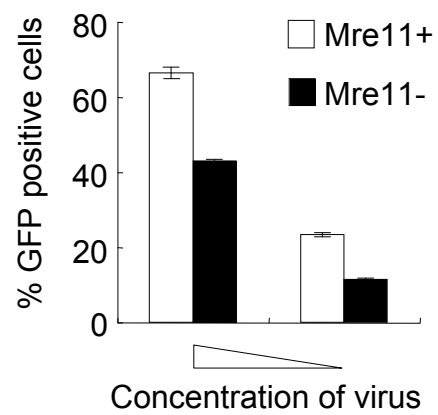

**Supplemental figure 1**

Supplement: Additional file 1 — Figure S1. Transduction efficiency of an MLV-based vector into cells deficient in DSB repair enzymes. Description: The transduction efficiency of the MLV-based vector was drastically decreased in DNA-PKcs-deficient cells and decreased in Mre11-deficient cells, but not altered in ATM- and NBS1-deficient cells. (A) ATM-deficient cells and ATM-complemented cells were transduced with the MLV-based vector encoding a GFP reporter. 2 days postinfection, the percentage of GFP-positive cells was determined by flow cytometry. (B-D) The influence of DNA-PKcs (B), NBS1 (C) and Mre11 (D) on transduction efficiency of the MLV-based vector was investigated by the same method as in (A). Error bars represent +/- SD. [file 1742-4690-6-114-S1.PDF]

A

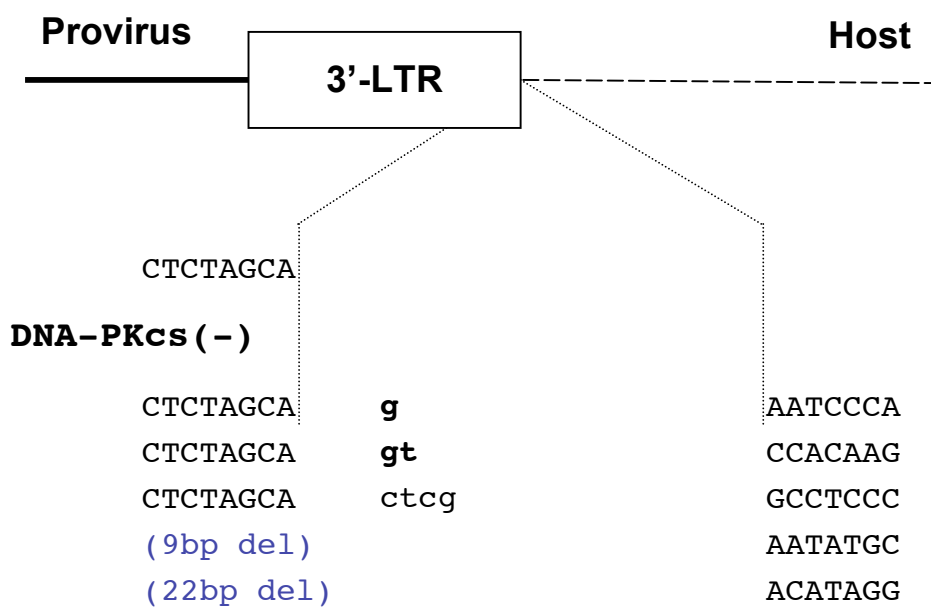

B

|                   | DNA-PKcs(-) |
|-------------------|-------------|
| <b>Insertions</b> | <b>3</b>    |
| Deletions         | 2           |
| Total junctions   | 153         |
| <i>P</i> value    | 0.17        |

**Supplemental figure 2**

Supplement: Additional file 2 — Figure S2. Abnormal 3' junctions of the HIV-1 provirus in DNA-PKcs-deficient cells. Description: (A) Junctions between the 3' end of the provirus and the host DNA were analyzed in DNA-PKcs-deficient cells transduced with an HIV-based vector. Inserted abnormal sequences are in lower case. Abnormal nucleotides corresponding to the GT dinucleotides processed by integrase are presented in bold. (B) The number of junctions with insertions and/or deletions. The P values under the table are for comparison of the number of junctions with only insertions or both insertions and deletions to that of MRC5SV cells in Table 1. [file 1742-4690-6-114-S2.PDF]
