# Supplementary material for: DNA double strand break repair enzymes function at multiple steps in retroviral infection
Source: Retrovirology. 2009 Dec 15;6:114. doi: 10.1186/1742-4690-6-114 (PMC2797772; doi:10.1186/1742-4690-6-114)
Supplement: Additional file 3 — Table S1. Primers for the sequence analyses around retroviral integration sites. [file 1742-4690-6-114-S3.PPT]

## Slide 1
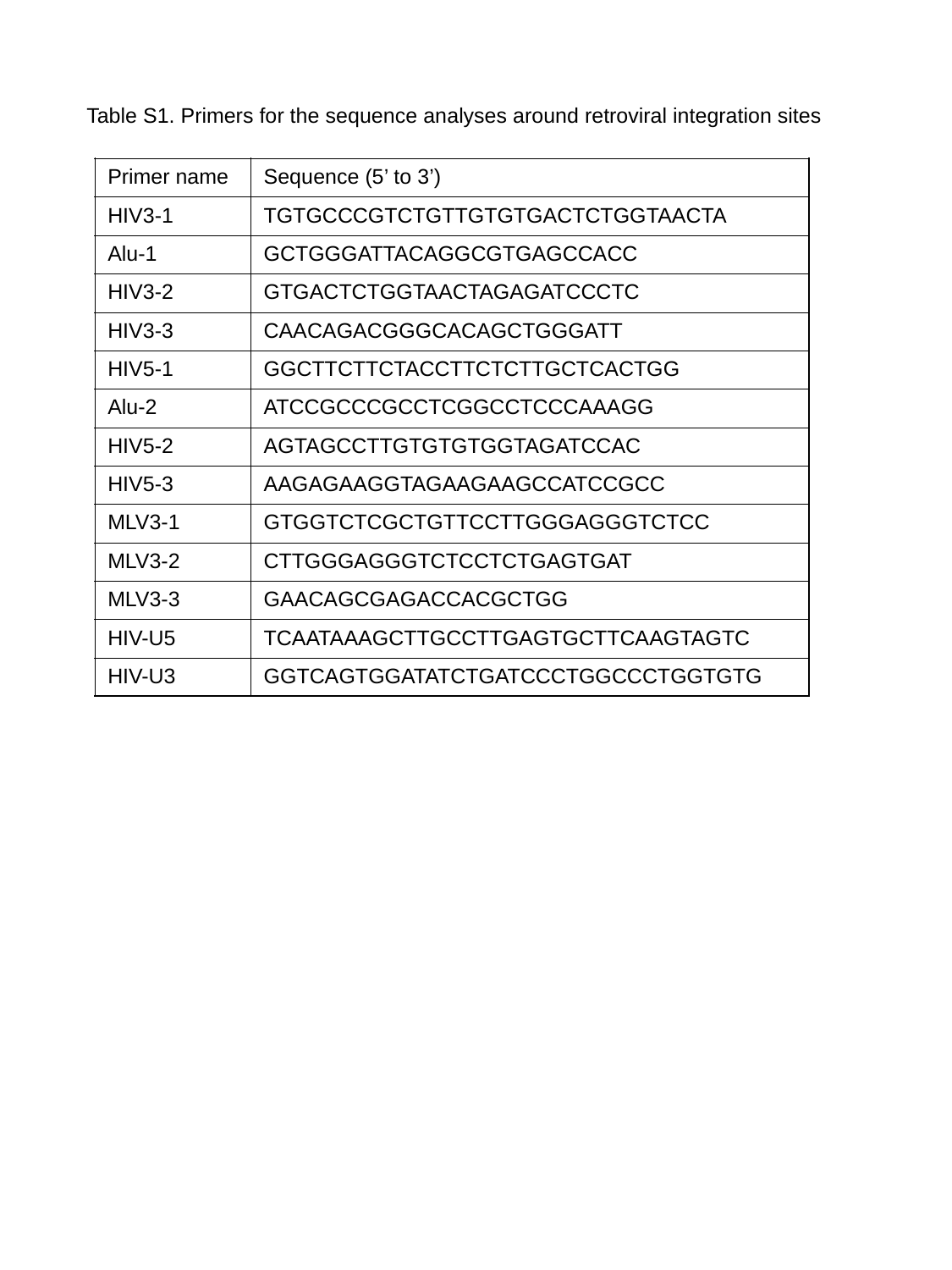

Table S1. Primers for the sequence analyses around retroviral integration sites
| Primer name | Sequence (5’ to 3’) |
| --- | --- |
| HIV3-1 | TGTGCCCGTCTGTTGTGTGACTCTGGTAACTA |
| Alu-1 | GCTGGGATTACAGGCGTGAGCCACC |
| HIV3-2 | GTGACTCTGGTAACTAGAGATCCCTC |
| HIV3-3 | CAACAGACGGGCACAGCTGGGATT |
| HIV5-1 | GGCTTCTTCTACCTTCTCTTGCTCACTGG |
| Alu-2 | ATCCGCCCGCCTCGGCCTCCCAAAGG |
| HIV5-2 | AGTAGCCTTGTGTGTGGTAGATCCAC |
| HIV5-3 | AAGAGAAGGTAGAAGAAGCCATCCGCC |
| MLV3-1 | GTGGTCTCGCTGTTCCTTGGGAGGGTCTCC |
| MLV3-2 | CTTGGGAGGGTCTCCTCTGAGTGAT |
| MLV3-3 | GAACAGCGAGACCACGCTGG |
| HIV-U5 | TCAATAAAGCTTGCCTTGAGTGCTTCAAGTAGTC |
| HIV-U3 | GGTCAGTGGATATCTGATCCCTGGCCCTGGTGTG |
